# In Vitro Anticancer Effect of Gedunin on Human Teratocarcinomal (NTERA-2) Cancer Stem-Like Cells

**DOI:** 10.1155/2017/2413197

**Published:** 2017-06-07

**Authors:** Luxmiga Tharmarajah, Sameera Ranganath Samarakoon, Meran Keshawa Ediriweera, Poorna Piyathilaka, Kamani Hemamamla Tennekoon, Kanishka Sithira Senathilake, Umapriyatharshini Rajagopalan, Prasanna Bandula Galhena, Ira Thabrew

**Affiliations:** Institute of Biochemistry, Molecular Biology and Biotechnology, University of Colombo, 90 Cumaratunga Munidasa Mawatha, 03 Colombo, Sri Lanka

## Abstract

Gedunin is one of the major compounds found in the neem tree* (Azadirachta indica)*. In the present study, antiproliferative potential of gedunin was evaluated in human embryonal carcinoma cells (NTERA-2, a cancer stem cell model) and peripheral blood mononuclear cells (PBMCs), using Sulforhodamine (SRB) and WST-1 assays, respectively. The effects of gedunin on expression of heat shock protein 90 (HSP90), its cochaperone Cdc37, and HSP client proteins (AKT, ErbB2, and HSF1) were evaluated by real-time PCR. Effects of gedunin on apoptosis were evaluated by (a) apoptosis associated morphological changes, (b) caspase 3/7 expression, (c) DNA fragmentation, (d) TUNEL assay, and (e) real-time PCR of apoptosis related genes (*Bax*,* p53, *and* survivin*). Gedunin showed a promising antiproliferative effect in NTERA-2 cells with IC_50_ values of 14.59, 8.49, and 6.55 *μ*g/mL at 24, 48, and 72 h after incubations, respectively, while exerting a minimal effect on PBMCs. Expression of HSP90, its client proteins, and* survivin* was inhibited and* Bax* and* p53* were upregulated by gedunin. Apoptosis related morphological changes, DNA fragmentation, and increased caspase 3/7 activities confirmed the proapoptotic effects of gedunin. Collectively, results indicate that gedunin may be a good drug lead for treatment of chemo and radiotherapy resistant cancer stem cells.

## 1. Introduction

Cancer stem cells (CSCs) are considered as initiators of tumor development and progression [1]. They possess stem cell characteristics such as proliferation, self-renewal, and differentiation into all cell types of the original tumor [2]. These CSCs are resistant to conventional chemotherapy, radiation therapy, and various natural and synthetic anticancer drugs, thus causing relapse of cancer after conventional treatments [3]. Therefore, it is essential to identify novel drug leads that not only target cancer descendant malignant cells, but also target CSCs without damaging normal cells [4]. As CSCs possess some unique dynamics and features, they can be targeted by several methods, including sensitization to natural or synthetic compounds, induction of differentiation into other cell types, and restricting self-renewal [5].

Among clinically approved anticancer drugs, approximately 60% are natural products derived from plants and microorganisms [6]. The hedgehog, Wnt/*β*-catenin, and Notch-mediated signaling pathways are considerd to be important in CSCs differentiation and self-renewal [7]. Several natural compounds have been reported to target these signaling pathways of CSCs. Cyclopamine, an alkaloid first isolated from* Veratrum californicum,* has been reported to target hedgehog signaling pathway [8]. Epigallocatechin gallate (EGCG), one of the main compounds in tea, has been reported to inhibit Wnt/*β*-catenin signaling pathway [9]. It has also been reported that Vitamin D and its analogs could inhibit Notch and Wnt/*β*-catenin signaling pathways [10]. Moreover, curcumin and piperine, well-known anticancer compounds, have also been reported to target breast CSCs [11].

Gedunin (tetranortriterpenoid) is one of the main chemical compounds found in the neem tree [12]. Recent studies have shown that gedunin can inhibit the proliferation of cancer cells including those of the prostate, ovary, and colon [13, 14]. It is also reported to be an Hsp90 (heat shock protein 90) inhibitor [15]. Furthermore, a recent in-silico study has revealed drug likeness of gedunin for *β*-catenin chain A in cancer stem cells [16]. However, antiproliferative and apoptotic effects of gedunin in cancer stem cells or cancer stem cell model have not yet been evaluated to date. NTERA-2 cells are highly pluripotent undifferentiated with cancer stem cell properties [17]. Therefore, the present study was carried out to evaluate the effectiveness of gedunin on human embryonal carcinoma (NTERA-2) cells as a cancer stem cell model.

## 2. **Methodology**

### 2.1. General

All the chemicals used in the study were purchased from Sigma-Aldrich (St. Louis, MO, USA) unless otherwise specified. NTERA-2 cell line and all the reagents used for cell culture were purchased from ATCC (Manassas, VA, USA). Gedunin was purchased from Santa Cruz Biotechnology (Santa Cruz, CA). Caspase 3/7 reagent and TUNEL assay kits were purchased from Promega (Promega Corporation, Madison, WI, USA). MESA Green qPCR Master Mix Plus for SYBR Assay was purchased from Eurogentec, Seraing, Liège, Belgium. WST-1 cell proliferation assay kit was purchased from Abcam (Cambridge, MA, USA).

### 2.2. Cell Culture

The NTERA-2 cells were cultured using Dulbecco's Modified Eagle's Medium (DMEM), supplemented with 10% fetal bovine serum (FBS), 50 IU/mL penicillin, and 50 *μ*g/mL streptomycin. Cells were incubated at 37°C with 5% CO_2_ and 95% humidity.

### 2.3. Sulforhodamine B (SRB) Assay

Prior to the SRB assay, NTERA-2 cells were grown in a 75 cm^3^ flasks. Upon confluency, cells were trypsinized and seeded into 96-well plates (5 × 10^3^ cells/well). After 24 h incubation, cells were treated with various concentrations (3.125, 6.25, 12.5, 25, and 50 *μ*g/mL) of gedunin and retinoic acid (positive control) dissolved in 0.1% dimethyl sulfoxide (DMSO) and incubated for 24, 48, and 72 h. Negative control for the experiment was 0.1% DMSO in DMEM medium. Then the cells were fixed with 20 *μ*L of 50% (trichloroacetic acid) TCA and incubated for further 1 h at 4°C. After incubation, cells were washed five times with tap water and stained with 50 *μ*L of 0.4% (w/v) SRB solution. After 20 min of incubation, SRB solution was poured off and unbound dye was removed by washing five times with 1% acetic acid. Bound SRB dye was then solubilized by adding 100 *μ*L of Tris base solution. The plates were shaken for 1 h and absorbance was read at 540 nm OD.

### 2.4. Preparation of Peripheral Blood Mononuclear Cells and WST-1 Cell Viability Assay

Cytotoxic effects of gedunin and positive control (retinoic acid) on normal human cells were evaluated by using human peripheral blood mononuclear cells. Collected blood was diluted with Hank's Buffered Salt Solution (HBSS) and layered onto Histopaque 1077. Blood was then centrifuged at 1000 rpm for 10 min. After centrifugation, opaque interface which contains mononuclear cells was seperated and washed with phosphate buffered saline (PBS). Cells were then counted and cultured (2 × 10^5^ cells/mL) in RPMI medium supplemented with 10% fetal bovine serum (FBS), 50 IU/mL penicillin, and 50 *μ*g/mL streptomycin in 24-well plates. Cells were then treated with different concentrations of gedunin and retinoic acid dissolved in 0.1% DMSO (3.125, 6.25, 12.5, 25, and 50 *μ*g/mL) and incubated for 24, 48, and 72 h. Negative control for the experiment was 0.1% DMSO in RPMI medium. WST-1 cell proliferation assay was then carried out according to manufacturer's instructions to determine the cytotoxic effects.

### 2.5. Acridine Orange and Ethidium Bromide (AO/EB) Staining

NTERA-2 cells (2.5 × 10^4^ cells/mL) were cultured on cell culture treated coverslips and incubated for 24 h. Subsequently, cells were exposed to different concentrations of gedunin dissolved in 0.1% DMSO (2.5, 5, and 10 *μ*g/mL) and incubated for 24 h. Negative control for the experiment was 0.1% DMSO in DMEM medium. After incubation, cells were fixed using 4% formaldehyde in the dark for 10 min. Cells were then washed with PBS and stained with ethidium bromide (100 *μ*g/mL) and acridine orange (100 *μ*g/mL) solutions (1 : 1 v/v). Excess dye was then washed off with PBS and cell morphology was observed using a fluorescence microscope (BX51 TRF, Olympus Corporation, Tokyo, Japan).

### 2.6. TUNEL Assay

Terminal deoxynucleotidyl transferase-mediated dUTP-biotin nick-end labeling (TUNEL) staining was carried out to confirm in situ chromatin fragmentation. Cells cultured on coverslips (2.5 × 10^4^ cells/mL) were treated with different concentrations of gedunin dissolved in 0.1% DMSO (2.5, 5, and 10 *μ*g/mL) and incubated for 24 h. Negative control for the experiment was 0.1% DMSO in DMEM medium. TUNEL staining was carried out according to manufacturer's instructions. Briefly, cells were fixed with 4% formaldehyde and permeabilized with 0.2% Triton X-100. Equilibration buffer (100 *μ*L) and TdT reaction mix to label DNA were added and incubated for further 60 min in a humidified chamber. Then coverslips were immersed in 2x SSC buffer for 15 min followed by immersing in 0.3% H_2_O_2_ for 3 min. Finally, labeled fragments were incubated with streptavidin horseradish peroxidase and visualized after diaminobenzidine (DAB) color development where dark brown spots confirmed apoptosis.

### 2.7. Caspase 3/7 Assay

NTERA-2 cells (2.5 × 10^4^ cells/mL) were cultured in 96-well cell culture plates and incubated for 24 h. After incubation, cells were treated with gedunin dissolved in 0.1% DMSO (2.5, 5, and 10 *μ*g/mL) and incubated for further 24 h. Negative control for the experiment was 0.1% DMSO in DMEM medium. Caspase 3/7 reagent (100 *μ*L) was then added to each well and incubated for 1 h in dark at 37°C. Luminescence of each well was measured by a plate reader.

### 2.8. DNA Fragmentation Analysis

DNA fragmentation analysis was carried out according to previously described methods [18, 19]. NTERA-2 cells (2.5 × 10^4^ cells/mL) were grown in cell culture flasks and incubated for 24 h. After incubation, flasks were treated with gedunin dissolved in 0.1% DMSO (5, 10, and 15 *μ*g/mL) and further incubated for 48 and 72 h. Negative control for the experiment was 0.1% DMSO in DMEM medium. At the end of the incubation period, cells were trypsinized and cell pellets were obtained. Cell pellets were then incubated for 60 min at 50°C in a 100 *μ*L of lysis buffer (100 mM Tris–HCl, pH 8, 100 mM NaCl, and 10 mM EDTA). Proteinase K (10 *μ*L, 20 mg/mL) was then added to cell lysates and incubated for 30 min at 50°C. After adding 3 *μ*L of 10 mg/mL RNase, the mixture was incubated for 2 h at 50°C. DNA was extracted in phenol chloroform-isoamyl alcohol and 1 *μ*g of isolated DNA from each sample was subjected to 2.0% of agarose gel electrophoresis and visualized under UV using a gel-doc system (Quantum-ST4 1100/20 M).

### 2.9. Nitrobluetetrazolium Reactive Oxygen Species Assay (ROS)

ROS measurement in gedunin treated NTERA-2 cells was carried out using previously described methods [20, 21]. Cells (2 × 10^3^) were cultured in 96-well cell culture plates and incubated for 24 h. After incubation cells were treated with gedunin (2.5, 5, and 10 *μ*g/mL) and incubated for further 24 h. Cells were then incubated with nitrobluetetrazolium (1 mg/mL) for 1 h at 37°C. After the incubation 100 *μ*L of DMSO was added to solubilize blue formazan, plates were shaken for 10 min at room temperature, and the absorbance was read at 620 nm.

### 2.10. RNA Isolation and Reverse Transcriptase Quantitative Polymerase Chain Reaction (RT-qPCR)

NTERA-2 (2.5 × 10^5^ cells/mL) cells were cultured in cell culture flasks and treated with gedunin dissolved in 0.1% DMSO (5 and 10 *μ*g/mL) for 24 h. Negative control for the experiment was 0.1% DMSO in DMEM medium. Following incubation, total RNA was extracted with TRIzol® reagent according to the manufacturer's instructions. For reverse transcription, extracted RNA (2 *μ*g) was mixed with 50 ng of random primers and the total volume was made up to 13.5 *μ*L with ultrapure water. Resulting mixture was incubated at 70°C for 5 min and, after incubation, it was kept on ice for 2 min. Complementary DNA (cDNA) was then synthesized by adding 2 *μ*L from the transcription mixture, 5 *μ*L of 5x MMLV-buffer, 5 *μ*L of 10 mM deoxynucleotide mixture, 25 units of RNasin, and 200 units of MMLV-reverse transcriptase in a reaction mixture of 25 *μ*L adjusted by adding ultrapure water and incubated at 37°C for 60 min. Real-time PCR was carried out in Stratagene Mx3000P using a master mix containing SYBR green (MESA Green qPCR Master Mix Plus for SYBR Assay). Primers used for real-time PCR are given in Table 1 and glyceraldehyde-3-phosphate dehydrogenase (GAPDH) was used as the internal housekeeping gene. All the real-time PCR reactions contained 2 *μ*L cDNA, 0.5 *μ*L of each primer (stock concentration 0.5 *μ*M), 12.5 *μ*L SYBR Green master mix, and ultrapure water (9.5 *μ*L). PCR amplification was performed in triplicate. PCR conditions (for HSP90, Cdc37, and HSF1) were as follows: denaturation step (95°C for 10 min) and 40 PCR cycles of three-step amplification (denaturation, 95°C for 30 sec; annealing, 60°C for 1 min; and extension, 72°C for 1 min). Annealing temperature was maintained at 58°C for 1 min for primers AKT, ErbB2, Bax, p53, and survivin. Results of real-time PCR were analysed using the formula 2^−ΔΔCt^ [22].

### 2.11. Statistical Analysis

GraphPad Prism 5 (GraphPad, Inc., La Jolla, CA, USA) software was used for statistical analysis in the study and results were expressed as the mean ± standard deviation of three individual experiments. One-way ANOVA with Tukey's post hoc test was used to compare groups and *p* < 0.05 was considered as statistically significant.

## 3. **Results and Discussion**

### 3.1. Effect of Gedunin on NTERA-2 Cells Proliferation

SRB assay indicated a dose and time dependant inhibition of NTERA-2 cell proliferation once treated with gedunin (Table 2). Cytotoxic effect of gedunin on normal peripheral blood mononuclear cells was not significant as indicated by WST-1 cell viability assay (Table 2). Retinoic acid was used as the positive control in antiproliferative cytotoxic evaluations. Gedunin exerted a potential antiproliferative effect on cancer stem cell model NTERA-2 cells when compared to the positive control retinoic acid. Several researchers have also shown antiproliferative effects of certain compounds such as rooperol and RC-6 ribonuclease in NTERA-2 cells in vitro [17, 23].

### 3.2. Effects of Gedunin on NTERA-2 Cell Apoptosis

Apoptosis is a cellular process which is responsible for the maintenance of homeostasis of organs and tissues [24]. Deregulation of apoptosis is mainly observed in cancer [25]. Several morphological and biochemical processes are associated with cellular apoptosis [26]. Morphological features associated with apoptosis include condensation of chromatin, reduction in cell volume and shape, nuclear fragmentation, membrane blebbing, and loss of membrane integrity [27]. Biochemical changes associated with apoptosis include activation of caspases, DNA fragmentation, breakdown of proteins, and recognition of cell membrane changes by phagocytic cells [28]. Sequential activation of caspases is one of the major hallmark features of apoptosis [29]. Two classes of caspases, namely, initiators and effectors, have been identified and caspase-3 and caspase-7 belong to the caspase effectors [30]. DNA fragmentation is also considered as a key feature in apoptosis [31]. It is mainly due to the activation of endonucleases which causes cleavage of DNA into internucleosomal fragments [32]. In the present study apoptosis in gedunin treated NTERA-2 cells was evaluated by morphological observations, DNA fragmentation, caspase 3/7 expression, and TUNEL assay.

#### 3.2.1. AO/EB Staining

**Figure 1:**
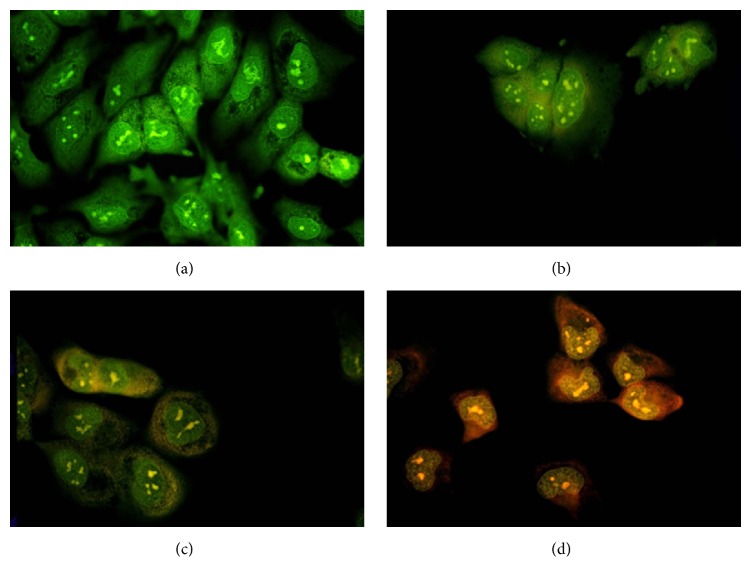
Morphological changes observed under florescence microscope in gedunin treated NTERA-2 cells after staining with AO/EB. (a) Untreated control, (b) cells treated with 2.5 *μ*g/mL of gedunin, (c) cells treated with 5 *μ*g/mL of gedunin, and (d) cells treated with 10 *μ*g/mL of gedunin.

AO/EB staining revealed morphological changes of apoptosis in gedunin treated NTERA-2 cells. Untreated cells were stained in green as these cells take up acridine orange (AO) whereas the cells undergoing apoptosis appeared in yellow/red as these cells take up ethidium bromide (EA) (Figure 1).

#### 3.2.2. CaspaseGlo 3/7 Activity

**Figure 2:**
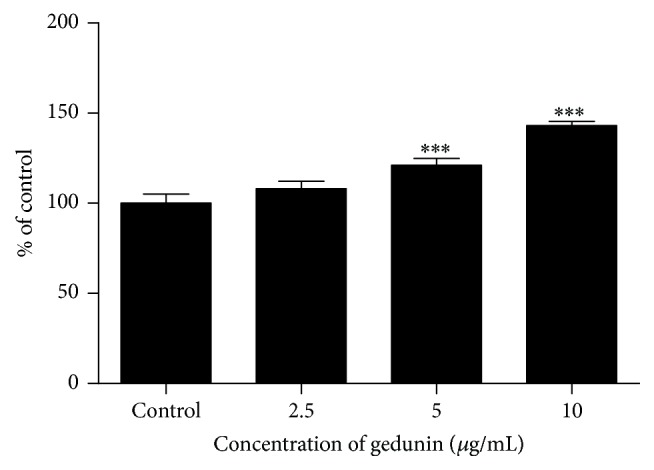
Activation of caspase 3/7 in NTERA-2 cells after exposure to gedunin for 24 h. ^*∗∗∗*^*p* < 0.0001.

Caspase 3/7 activity significantly increased in NTERA-2 cells after treatment with gedunin for 24 h. Significant (*p* < 0.0001) increase in caspase 3/7 was observed at two doses (5 and 10 *μ*g/mL) tested compared to the untreated control (Figure 2). Fold changes observed in caspase 3/7 activity at 2.5, 5, and 10 *μ*g/mL doses of gedunin treated NTERA-2 cells were 1.08, 1.2, and 1.41, respectively.

#### 3.2.3. DNA Fragmentation

**Figure 3:**
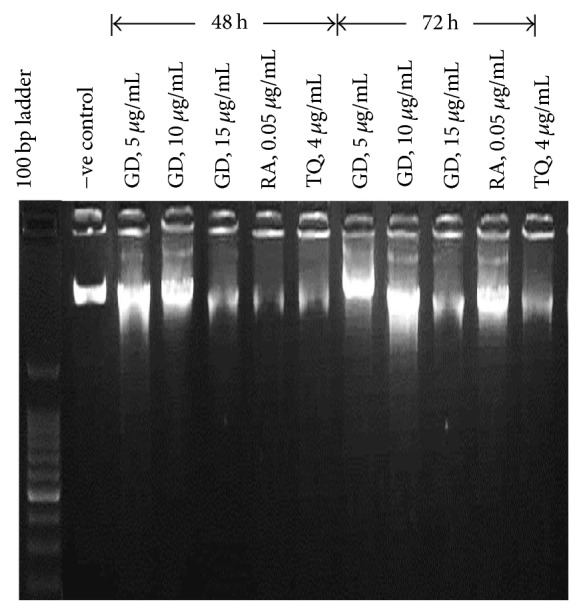
DNA fragmentation in gedunin (GD) treated NTERA-2 cells. RA and TQ are retinoic acid and thymoquinone (positive controls), respectively. −ve control is untreated cells (0.1% DMSO).

DNA fragmentation was observed in NTERA-2 cells exposed to gedunin for 48 and 72 h. A smeared laddering pattern of DNA was observed in the samples treated with 5, 10, and 15 *μ*g/mL of gedunin and positive controls (retinoic acid and thymoquinone). The untreated (control) samples did not show any laddering pattern (Figure 3).

#### 3.2.4. TUNEL Assay

**Figure 4:**
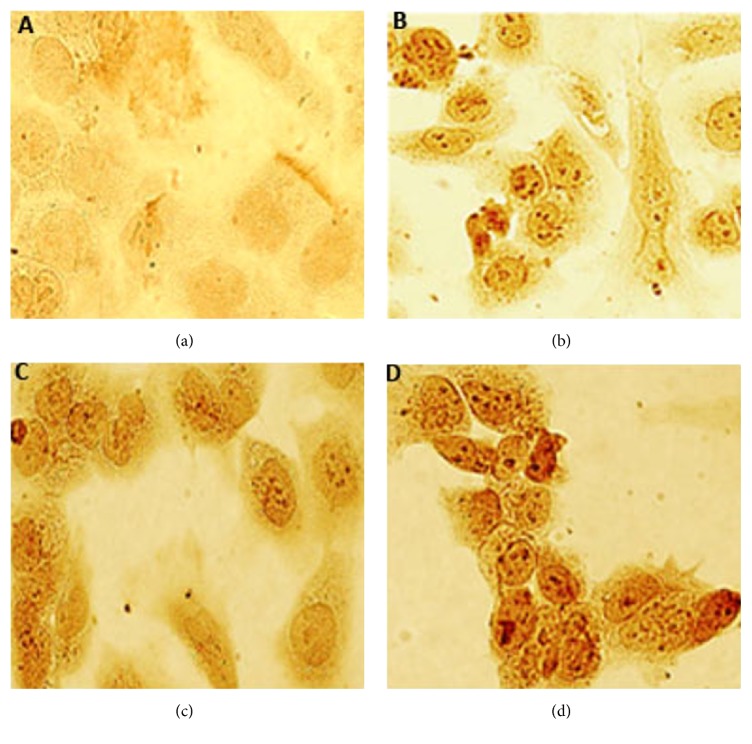
Apoptotic NTERA-2 cells after exposure to gedunin evaluated by TUNEL assay. (a) Untreated control, (b) cells treated with 2.5 *μ*g/mL of gedunin, (c) cells treated with 5 *μ*g/mL of gedunin, and (d) cells treated with 10 *μ*g/mL of gedunin.

Terminal deoxynucleotidyl transferase (TdT) dUTP Nick-End Labeling (TUNEL) assay has been used to detect apoptotic DNA degradation in late apoptosis. In this method, nicks in degraded DNA are identified by TdT. Gedunin caused increase in the number of TUNEL-positive nuclei at all the doses tested (2.5, 5, and 10 *μ*g/mL) in NTERA-2 cells. TUNEL-positive nuclei were not visible in untreated controls (Figure 4).

### 3.3. Gedunin Caused ROS Generation in NTERA-2 Cells

**Figure 5:**
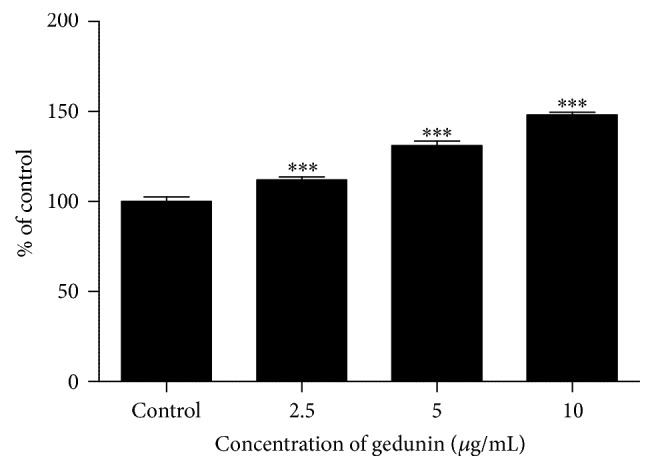
Reactive oxygen species (ROS) levels detected in gedunin treated NTERA-2 cells after 24 h incubation. ^*∗∗∗*^*p* < 0.0001.

Significant (*p* < 0.0001 and *p* < 0.001) increase in ROS production was observed in gedunin treated NTERA-2 at 24 h after incubation (Figure 5). Accumulation of ROS plays an important role in induction of apoptosis and cell cycle arrest in cancer cells [33]. Natural compounds such as curcumin, epigallocatechin gallate, parthenolide, quercetin, some phenolic lipids, and quercetin are reported to generate ROS and cause apoptosis in cancer cells [34]. Therefore it is likely that generation of ROS after treatment with gedunin causes apoptosis in NTERA-2 cells.

### 3.4. Effects of Gedunin on the Expression of Apoptotic Related and Heat Shock Protein (HSP90) and Its Client Protein Genes

**Figure 6:**
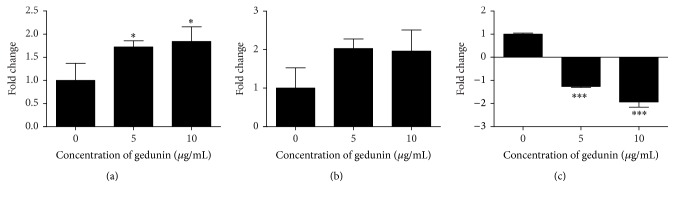
Effects of gedunin on the expression of apoptotic (*p53* and* Bax*) and antiapoptotic* (survivin)* related genes. (a)* p53*, (b)* Bax,* and (c)* survivin*. ^*∗*^*p* < 0.05, ^*∗∗∗*^*p* < 0.0001 when compared to untreated controls.

**Figure 7:**
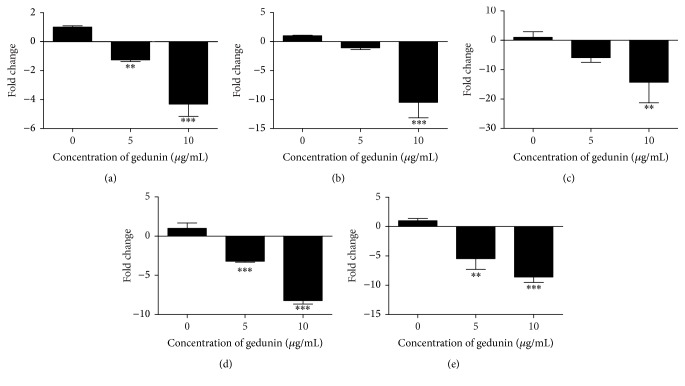
Effects of gedunin on the expression of heat shock protein (HSP90), its cochaperon (Cdc37), and client proteins (ErbB2, HSF1, and AKT). (a) HSP90, (b) Cdc37, (c) HSF1, (d) AKT, and (e) ErbB2. ^*∗∗*^*p* < 0.05, ^*∗∗∗*^*p* < 0.0001 when compared to untreated controls.

Several genes have been reported to exert proapoptotic and antiapoptotic effects in cancer cells [35].* Bax* and* p53* genes play a pivotal role in apoptosis [36].* Bax* is a proapoptotic gene which is known to induce apoptosis in cancer cells [37].* p53* is a tumor suppressor gene with various cellular functions [38]. Induction of apoptosis is one of its main functions [39]. It has been reported that* p53* can also regulate the antiapoptotic gene* survivin* [40]. In the present study, significant (*p* < 0.05) upregulation of* p53* was observed in NTERA-2 cells at both doses (5 and 10 *μ*g/mL) of gedunin tested. Upregulation of* Bax* was also observed in gedunin treated NTERA-2 cells at both the doses tested. However, regulation of* Bax *at 8 *μ*g/mL showed a slight reduction compared to the dose of 4 *μ*g/mL (Figure 7). Gedunin also caused significant (*p* < 0.0001) downregulation of proapoptotic gene* survivin* at both the doses tested in NTRTA-2 cells (Figure 6).

In the present study significant (*p* < 0.001 and *p* < 0.0001) downregulation of HSP90 and one of its cochaperones Cdc37 was observed at both the doses (5 and 10 *μ*g/mL) tested in gedunin treated NTERA-2 cells (Figure 7). Significant downregulation (*p* < 0.001 and *p* < 0.0001) of HSP90 client proteins (ErbB2, HSF1, and AKT) was also observed in gedunin treated NTERA-2 cells after 24 h incubation (Figure 7). This observation reveals that gedunin can inhibit the expression of HSP90 and its cochaperon Cdc37 through its client proteins (ErbB2, HSF1, and AKT) in NTERA-2 cells.

Heat shock proteins (HSP) play an important role in cancer development and progression [41]. These proteins are overexpressed in many cancers and they have been mainly targeted in cancer therapy [42]. HSP90 is one of the heat shock proteins reported to function as multichaperone complexes by combining with different cochaperones (Cdc37, p23, Hop, PP5, SGT1, Aha 1, etc.) affecting binding ability of HSP90 to client proteins (AKT, KIT, ErbB2, CDK 4, HSF1, Apaf-1, MMp2, etc.) [43]. Recent studies have confirmed that some natural compounds such as curcumin, quercetin, and taxifolin can inhibit HSP expression cancer cells [44]. A study carried out by Brandt et al. (2008) has demonstrated antiproliferative effects of gedunin and its derivatives in MCFC-7 and SKBR-3 breast cancer cells [45]. Patwardhan et al. (2013) have also demonstrated that gedunin can inhibit the HSP90 chaperone machine through inhibition of its cochaperone p23 [12]. AKT is a signaling molecule/kinase which participates in various cellular functions including apoptosis, cell proliferation, metabolism, and cell migration [46]. Inhibition of AKT action is reported to be essential in cancer cell apoptosis [47]. AKT is reported to be overexpressed in cancer cells and stabilized after interacting with HSP90 and one of its cochaperones, Cdc37 [48]. Hence, inhibition of HSP90 and its cochaperone Cdc37 through AKT is likely to cause apoptosis in cancer cells [49]. Human epidermal growth receptor 2 (HER-2/ErbB2) is strongly associated with poor prognosis in cancer [47–50]. It has been reported that ErbB2 activates client protein HSF1 and thereby controls the action of HSP90 in cancer cells [51]. Induction of apoptosis through AKT with the involvement of ErbB2 and HSP90 has also been reported in several studies [52]. Induction of apoptosis via HSP90 inhibition in response to some selected natural compounds has also been demonstrated in several studies [52, 53]. Apoptotic related genes (*Bax, p53,* and* survivin*) to HSP inhibition pathway/s have also been reported. Therefore, according to the results obtained from apoptosis related genes and HSP expression, it is clear that gedunin mediates apoptosis via inhibition of HSP90 and its client proteins in NTERA-2 cells. Even though this study presents downregulation of HSP90 client proteins by gedunin at transcription level, there is no supportive evidence in literature regarding inhibition of client proteins of HSP90 at transcription level by other HSP90 inhibitors. Therefore further investigations are needed to confirm regulation of client proteins of HSP90 by gedunin at transcriptional, translational, and posttranslational levels.

## 4. **Conclusion**

Based on the results obtained in the present study, gedunin has the potential to exert antiproliferative effect in NTERA-2 cells compared to peripheral blood mononuclear cells. Inhibition of heat shock protein expression (HSP90), its cochaperone and,client proteins appears to be one of the mechanisms used by gedunin to exert antiproliferative and apoptotic effects in the cancer stem cell model, NTERA-2 cells.

## Figures and Tables

**Table 1:** List of primers used for real-time PCR.

ID	Forward primer (5′-3′)	Reverse primer (5′-3′)	Size
HSP90	CGCTCCTGTCTTCTGGCTTC	TGGTATCATCAGCAGTAGGGTCA	117
Cdc37	GGGAGCAGAAAGACAAGACC	GTGGACGTTGTCTGACAGGT	110
HSF1	GCCTTCCTGACCAAGCTG	AAGTACTTGGGCAGCACCTC	134
AKT	TCTATGGCGCTGAGATTGTG	CTTATTGTGCCCGTCCTTGT	113
ErbB 2	AGCCGCGAGCACCCAAGT	TTGGTGGGCAGGTAGGTGAGTT	147
Bax	TCCAGGATCGAGCAGGGCGAA	CGATGCGCTTGAGACACTCGCT	109
p53	TCTGGCCCCTCCTCAGCATCTT	TTGGGCAGTGCTCGCTTAGTGC	369
Survivin	TGGCCGCTCCTCCCTCAGAAAA	GCTGCTGCCTCCAAAGAAAGCG	190
GAPDH	GGCATTGCCCTCAACGACCAC	ACATGACAAGGTGCGGCTCCCTA	283

**Table 2:** IC_50_ values (*μ*g/mL) of gedunin in NTERA-2 and peripheral blood mononuclear cells at 24, 48, and 72 h after incubation periods.

Cell type	Gedunin			Retinoic acid		
	24 h	48 h	72 h	24 h	48 h	72 h
NTERA-2 cells	14.59	8.49	6.55	14.36	9.167	6.872
Peripheral blood mononuclear cells	>100	>100	>100	>100	>100	>100
